# Systematic and meta-analysis of *Mycobacterium avium *subsp.* paratuberculosis* related type 1 and type 2 diabetes mellitus

**DOI:** 10.1038/s41598-022-08700-4

**Published:** 2022-03-17

**Authors:** Temitope C. Ekundayo, Ayodeji O. Falade, Bright E. Igere, Chidozie D. Iwu, Mary A. Adewoyin, Tosin A. Olasehinde, Oluwatosin A. Ijabadeniyi

**Affiliations:** 1 Department of Biotechnology and Food Science, Durban University of Technology, Steve Biko Campus, Health Services, 121 Steve Biko Rd, Musgrave, Berea, Durban, 4001 South Africa; 2 Department of Biological Sciences, University of Medical Sciences, Ondo City, Ondo State Nigeria; 3 Biotechnology, Computational Biochemistry and Phytomedicine Research Group, Department of Biochemistry, University of Medical Sciences, Ondo City, Ondo State Nigeria; 4 Department of Microbiology and Biotechnology, Western Delta University, Oghara, Delta State Nigeria; 5 School of Health Systems and Public Health, Faculty of Health Sciences, University of Pretoria, Pretoria, South Africa; 6 Department of Biological Sciences, Anchor University, Ayobo Road, Ipaja, Lagos, Nigeria; 7 Nutrition and Toxicology Division, Food Technology Department, Federal Institute of Industrial Research Oshodi, Lagos, Nigeria; 8 Discipline of Microbiology, School of Life Sciences, University of Kwazulu-Natal, Westville campus, Durban, South Africa

**Keywords:** Biochemistry, Immunology, Autoimmunity, Pathogens, Microbiology, Policy and public health in microbiology

## Abstract

Global increase in diabetes (DM) prevalence necessitated the need to establish the association between DM and environmental triggers including MAP (*Mycobacterium avium *subsp.* paratuberculosis*) that have been postulated to play a role in DM etiopathology for effective management. The present investigation aimed to assess the odds ratio (OR) presenting the association between MAP and DM. MAP-related DM studies were systematically retrieved from 6 databases until 31 September 2021 according to PRISMA principles for data abstraction. The abstracted dataset was fitted to the fixed-effects (FE) and random-effects (RE) models using the Mantel–Haenszel approach. Sixteen studies involving 2072 participants (1152 DM patients (957 type 1 diabetes mellitus (T1DM) & 195 type 2 diabetes mellitus (T2DM)) and 920 healthy controls) met the inclusion criteria. Results revealed a significant association between anti-MAP antibodies (abs) seroprevalence and T1DM (FE: OR 7.47, 95% CI 5.50**–**10.14, *p* value < 0.0001; RE: OR 7.92, 95% CI 4.39**–**14.31, *p* < 0.0001) and MAP DNA with T1DM (FE: OR 4.70 (95% CI 3.10**–**7.13, *p* value < 0.0001), RE: OR 3.90 (95% CI 0.93–16.38, *p* value = 0.06)). Both anti-MAP abs and MAP DNA based meta-analyses had medium heterogeneity (I^2^ = 47.2–61.0%). Meanwhile, no significant association between MAP and T2DM (FE: OR 1.13, 95% CI 0.54–2.37, *p* value = 0.74; RE: OR 1.19; 95% CI 0.34–4.12, *p* value = 0.69), its OR magnitude exceeded 1 and prediction interval (0.09–15.29) suggest possibility of association between the duo in the future. The leave-one-out sensitivity analysis depicts a robust meta-analysis in all cases. In conclusion, the study manifests a positive association between MAP and T1DM, highlighting that MAP prevention and environmental control would indubitably revolutionize T1DM management. Also, its projects possible link between MAP and T2DM as more data becomes available. However, it remains elusive whether MAP triggers T1/T2DM or a mere comorbidity in T1/T2DM. Epidemiological activities to fill the global/regional data gaps on MAP-related T1DM and T2DM are advocated in order to assess the burden of MAP-related DM and improve their clinical management.

## Introduction

*Mycobacterium avium subsp. paratuberculosis* (MAP) is a Gram positive, acid-fast, obligate intracellular and rod-shaped bacterium with a thick cell wall responsible for its robustness within a host cell and the environment^1,2^. MAP causes paratuberculosis (ParaTB), a chronic mycobacteriosis of ruminants. ParaTB/MAP is globally widespread and impacts gravely on the economy, animal well-being and public health^3^ and can manifest as an isolated clinical case or as an outbreak partly contingent on how long it is present within a herd^3^. Popularly known to infect dairy cattle, sheep and goats, MAP induced chronic enteric inflammation in other monogastric animals like pigs, dogs and sub-human primates including cotton-top tamarins, baboons, macaques and gibbons has been reported^4^. Infected animals regularly shed this pathogen via their faeces, milk and colostrum making human exposure possible^5^.

Additionally, MAP has been isolated from surface water^6,7^, soil^6^, cattle-based manure applied to agricultural land^8,9^, municipal tap water^8,9^, and infant formula made from pasteurized milk^10^, increasing the routes through which MAP could be transmitted to human. Several human diseases have been attributed to MAP and they include Crohn’s disease^11^, sarcoidosis and Blau syndrome^1^, type 1 diabetes^12^, Hashimoto’s thyroiditis^13^, and multiple sclerosis^14^. However, data on prevalence/epidemiology of MAP infection in human is generally limited compared to animals. The prevalence of MAP DNA has been reported to be > 90% in Crohn’s disease^15^, 42% in MS (multiple sclerosis) vs. 12.5% HC (*p* = 0.0008)^14^ and 27.5% MS vs. 6.3% HCs (*p* < 0.0001)^16^.

Diabetes (DM) is a chronic metabolic disease characterized by high blood glucose level, which occurs as a result of deficit in insulin and/or insulin resistance^17^. As one of the top five causes of death globally, the 463 million people reckoned to be diabetic worldwide in 2019 was projected to increase to 700 million by 2045^18^. The approximately 19 million adults living with diabetes in Africa are estimated to rise to 45 million by 2045^18^. Also, 1 in every 5 of the people who are older than 65 years are diabetic^18^. DM is categorized into three main types: type-1-diabetes mellitus (T1DM), type-2-diabetes mellitus (T2DM) and gestational diabetes mellitus^19^. T1DM is regarded as an autoimmune disease, with different environmental agents including MAP as potential triggers in people who are genetically at risk of developing T1DM^20^. Several studies have linked early dietary exposure to cow milk proteins and the increased risk of TIDM^21–23^on the observation that children at risk of TIDM exclusively fed on breast milk > 6 months had reduced likelihood to have TIDM later in life compared to their counterparts weaned onto cow-based formula milk at an earlier age^24^. The relationship between MAP and DM (T1DM) is not fully known. However, MAP has been found to have certain epitopes which are functionally, immunologically and structurally similar to that of the human host cells. This phenomenon is regarded as “epitope mimicry”^2^. The actual association between MAP and DM require evidential elucidation. This study was therefore conceived to assess the association between MAP and DM considering the odds ratio (OR) of MAP infection in DM as the outcome of interest.

## Materials and methods

### Study design and article identification

Studies (research articles) that elucidated MAP infections in diabetes mellitus (T1DM and T2DM) were systematically retrieved from ProQuest, Scopus, PubMed, EBSCOhost, Google Scholar, and Web of Science (WoS). The identifier-algorithm applied was ‘paratuberculosis AND (T1D* OR diabet* OR T2D*)’ or ‘'Johne’s disease' AND (T1D* OR diabet* OR T2D*)’ as title-specific search in the databases without lingual, regional and spatial–temporal bounds. For Google Scholar, the modified identifier-Boolean, ‘allintitle: paratuberculosis T1D OR diabetes OR diabetic OR T2D -review -case -cases -animal -mice -mouse’ was utilized. All records were retrieved, imported and de-duplicated in EndNote 20, and processed following the “Preferred Reporting Items for Systematic Reviews and Meta-analyses (PRISMA) guidelines”^25^. Documents were firstly retrieved on 20 September 2021 and with continuous email-alert-tracking until 31 September 2021 in the 6 databases. Summary of the retrieval and PRISMA pre-processing of the articles is given in Fig. S1 and full identifier-Boolean for each database is presented in supplementary material S1. The corresponding author (E.T.C.) performed the search, screened the abstracts and titles for inclusion in this study.

### Inclusion criteria

The studies of interest were those that definitively investigated MAP infections (anti-MAP antibodies (abs) or MAP DNA) in diabetic conditions (T1DM and T2DM) and provided the following information: number of diabetic patients versus (vs.) healthy controls (HCs), number (or prevalence) of anti-MAP + antibody/MAP DNA + diabetic patients vs. number (or prevalence) of MAP + /MAP DNA + HCs, and full description of MAP detection techniques. Where more than one anti-MAP abs were targeted in ELISA, prevalence based on positivity to at least one of the antigen/peptides studied was recorded. Cohort studies were not available on the MAP-related MS conditions. The qualities of the included studies were assessed according to Newcastle–Ottawa Quality Assessment Form for Case–Control Studies (Supplementary material S1) and discussed in term of percentage of the studies meeting each quality/sub-quality item.

The data were extracted in a predesigned excel form by 5 authors (T.A.O., A.O.F., B.E.I., M.A.A., and C.D.I) and two authors (E.T.C. and A.O.F.) validated the extracted data and resolved any case of variance by discussion.

### Data extraction

The data targeted and extracted were first author, year of publication, nation, number of diabetic patients, number of HCs, number (or prevalence) of anti-MAP + (MAP DNA +) in diabetic patients, number (or prevalence) of anti-MAP + (MAP DNA +) in HCs, and MAP testing techniques (PCR/ELISA). In addition, age, definition of DM and gender data of participants were also recorded.

### Statistical analysis

The meta-analysis was conducted in R version 4.1.1. The functionalities of the following r packages: meta version 4.18-2^26^, PerformanceAnalytics version 2.0.4^27^, dmetar version 0.0.9000^28^, and metafor version 3.0-2^29^ were utilized. The MAP DNA and anti-MAP abs data in diabetic patients and HCs were separately fitted to random-effects (RE) and fixed-effects (FE) models using the Mantel–Haenszel approach^30–32^. For τ^2^ estimation in both models, the Sidik-Jonkman estimator with Hartung-Knapp adjustment was used^33^. Further, heterogeneity between studies was quantified using Q-statistic and I^2^-statistic at a significant heterogeneity threshold of an I^2^ > 75%^34^.

Sensitivity analysis of the meta-analysis was performed using the leave-one-out-method^35^. Publication bias was determined using funnel plot and Egger’s tests^36^ when the pooled studies ≥ 10^37^. In addition, P-curve analysis was performed to test whether the effect size from the meta-analysis is not due to publication bias/p-hacking^38,39^.

## Results

The present study investigated the connection between MAP and diabetes mellitus. 125 MAP-related articles were identified from the 6 databases (Figure S1). Following de-duplication, 96 duplicates and 7 others (4 theses, 2 reviews, and 1 Book chapter) were removed after titles and abstracts were screened. The full-text of the remaining 22 articles were reviewed for data extraction. Four additional articles were discarded including 3 hypothesis articles unsupported with data and 1 article lacking relevant data for inclusion. A total of 18 eligible articles were found involving 2 prevalence studies and 16 case–control studies. Cohort studies were not available on MAP-related MS conditions. However, only the 16 case–control studies (1152 DM patients (957 T1DM & 195 T2DM) and 920 HCs) were considered and meta-analysed. The 16 studies reported MAP-related T1DM^12,40–53^ and among which, 4 reported MAP-related T2DM studies^50,52,54,55^ in addition. The included studies were conducted in Asia (Iran, 1/16), Europe (Italy, 14/16) and North America (USA, 1/16) (Table 1).

**Table 1:** Explanatory characteristics of the MAP-related diabetes studies included in the meta-analysis.

S/n	Author	MAP + DM patients	DM patients	MAP + HCs	HCs	DM patients age (years)	HC age (years)	DM*	M/F_dm_\M/F_hc_	Method	DM type	Nation	Continent
1	Bitti et al.^34^ 2012	76//30	247	5//5	110	nt	nt	nt	nt	ELISA//PCR	T1DM	Italy	Europe
2	Niegowska et al.^47^ 2016b	26	32	4	42	^×^8.90 ± 3.52	^×^6.90 ± 3.55		19/13\	ELISA	T1DM	Italy	Europe
3	Masala et al.^41^ 2011	9//19	34	4//13	63	34.5 ± 7.7	38.5 ± 12.8	nt	nt	ELISA//PCR	T1DM	Italy	Europe
4	Masala et al.^42^ 2012	23	50	4	51	^×^33.5 ± 7	^×^36.36 ± 7	AADC	nt	ELISA	T1DM	Italy	Europe
5	Pinna et al.^43^ 2013	6	23	2	39	nt	nt	nt	nt	ELISA	T1DM	Italy	Europe
6	Pinna et al.^44^ 2014t1	11	62	3	81	48.6 ± 13.5	48 ± 12	FPGL/ GPL	31/31\39/42	ELISA	T1DM	Italy	Europe
7	Sechi et al.^45^ 2008a	32	59	2	59	36 ± 10.70	37 ± 12.29	nt	32/27\36/23	ELISA	T1DM	Italy	Europe
8	Sechi et al.^39^ 2008b	29	46	8	50	nt	nt	nt	nt	PCR	T1DM	Italy	Europe
9	Masala et al.^38^ 2014	25	107	9	100	^×^9.4 ± 5	^×^9.3 ± 3.5	AADCC	27/32\32/28	ELISA	T1DM	Italy	Europe
10	Rosu et al.^46^ 2009t1	27	57	10	79	nt	nt	nt	29/28\	ELISA	T1DM	Italy	Europe
11	Niegowska et al.^40^ 2016zn	38	54	7	42	9.42 ± 3.84	6.94 ± 3.58	**TAP**	27/27\	ELISA	T1DM	Italy	Europe
12	Shariati et al.^35^ 2016	9//15	29	9//0	29	^×^17.2	^×^16.3		7/22\	ELISA//PCR	T1DM	Iran	Asia
13	Cossu et al.^12^ 2011t1	21	43	2	48	35.5 ± 11.3	37.0 ± 12.85	nt	21/21\30/18	ELISA	T1DM	Italy	Europe
14	Noli et al.^36^ 2021	13	45	2	45	12.6//7.8**	11.8	AADC	46/25\16/29	ELISA	T1DM	Italy	Europe
15	Naser et al.^48^ 2013t1	3	10	3	6	nt	nt	nt	7/5\1/5	PCR	T1DM	USA	North America
16	Paccagnini et al.^39^ 2009	33	59	18	76	^z^18–94	nt	nt	nt	PCR	T1DM	Italy	Europe
17	Pinna et al. ^44^ 2014t2	6	80	3	81	^×^66.6 ± 74	^×^48 ± 12	sm	51/29\39/42	ELISA	T2DM	Italy	Europe
18	Naser et al.^48^ 2013t2	1	2	3	6	nt	nt	nt	nt	PCR	T2DM	USA	North America
19	Rosu et al.^46^ 2009t2	4	57	10	79	nt	nt	nt	36/21\	ELISA	T2DM	Italy	Europe
20	Cossu et al.^12^ 2011t2	5	56	2	48	^×^66.0 ± 8.71	^×^37.0 ± 12.85	nt	36/20\30/18	ELISA	T2DM	Italy	Europe

AADC: American Association of Diabetes criteria, ^x^ = median age instead of mean age (or, z = age range) reported in the column; nt = not reported; **dm with onset of DM; FPGL = fasting plasma glucose level > 126 mg/dL; GPL PGL = plasma glucose level > 200 mg/dL 2 h. after a 75-g oral glucose load in a glucose tolerance test (as defined by the WHO); TAP = T1D-related autoantibodies positivity.

Table S1 presents the qualities of the included studies. Based on selection quality of cases, 37.5% of the studies accompanied case definition adequately with laboratory diagnosis/validation (item 1a)^42,44,49–51,56^ while 68.8%^41–43,45,47,52,54,57–59^ relied on hospital records for case definition (item 1b); in term of representativeness of the cases, 93.8%^42–45,47,50–52,54,57–61^ represent representative series of cases (item 2a), 6.1%^41^ had or did not stated potential for selection biases (item 2b). On selection of controls, 18.8% of the studies^51,54,58^ were based on community controls (item 3a), while 68.8% studies were hospital-based controls^42–44,47,50,52,57–61^ (item 3b). All the studies (100%) defined controls based on no history of DM^12,41–45,47,49–52,54,57–61^ (item 4a). No further efforts to assay/validate the true non-DM statuses of the controls via diabetic diagnosis test in most cases. For quality based on comparability of design and analysis to control for confounders, 81.3% of the studies^12,41–45,47,49,50,52,58,60,61^controls for age and 25.0%^42,46,49,57^ further control for other sources of confounders. For exposure quality, ascertainment of MAP exposure in DM cases and controls was based on anti-MAP antibodies (abs)/MAP DNA diagnostic approach (PCR) in all the studies (100%; item 1a) and the same for both DM cases and controls (100%, item 1b)^12,41–45,47,49–52,54,57–61^, and non-response rate was generally not reported but the same in both cases and controls in 12.5%^45,51^ of the studies (item 3a) and different between the groups with no description in 6.3%^49^ (item 3c).

**Figure 1:**
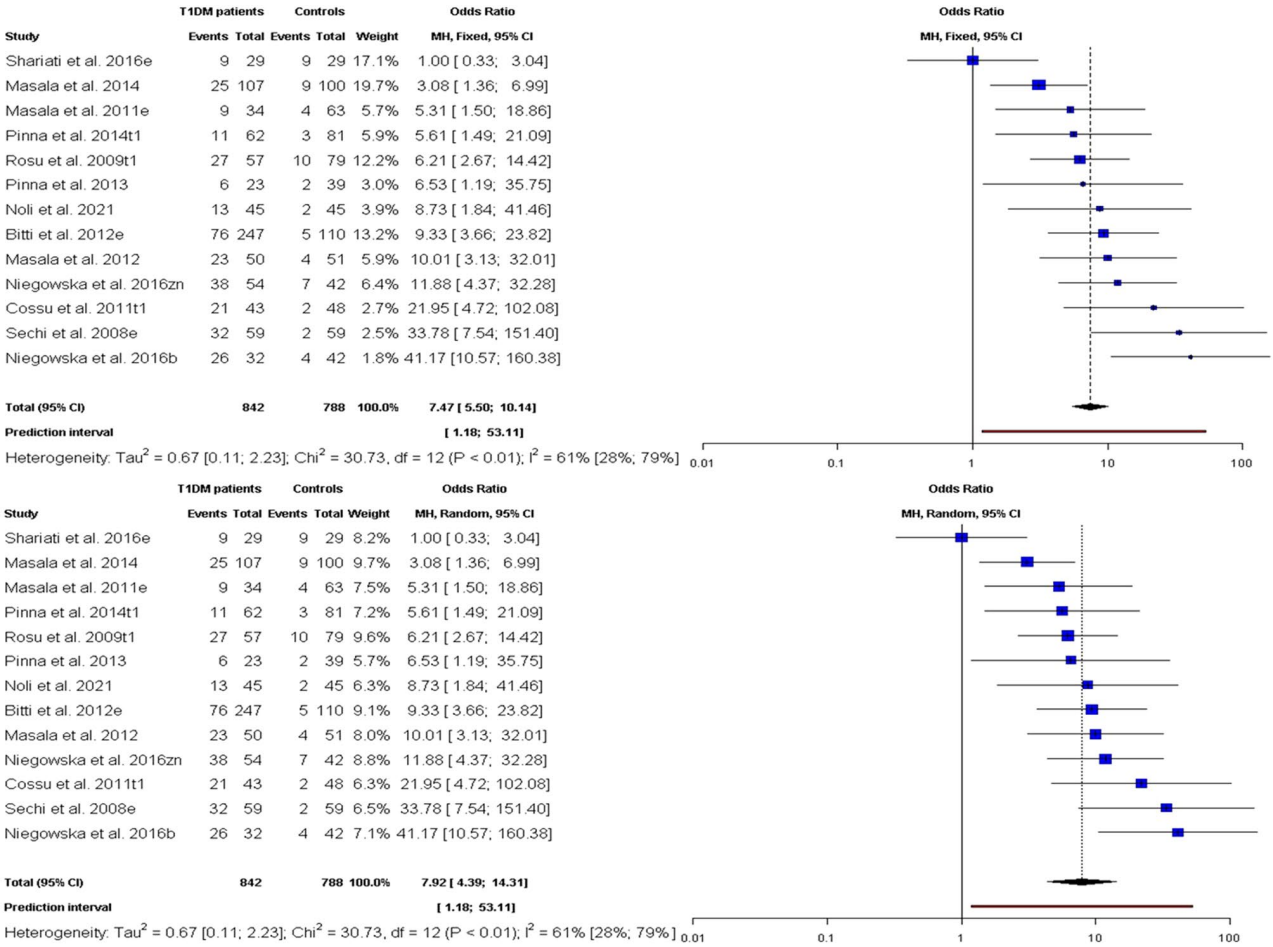
Forest plots for meta-analysis of the risk of anti-MAP (abs) infections linked with T1DM.

**Figure 2:**
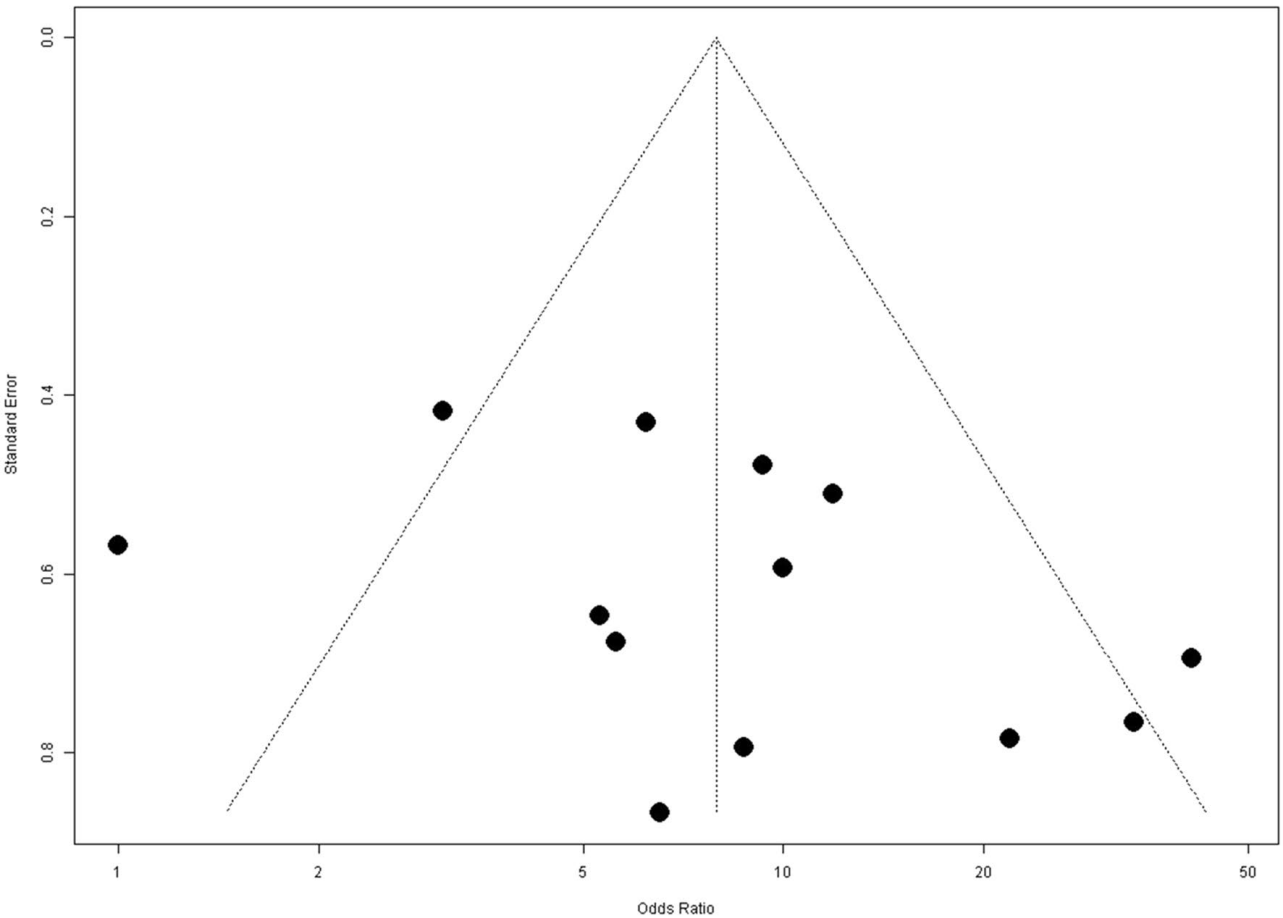
Funnel plot for anti-MAP abs meta-analysis. Eggers' test of the intercept (Eggers' test (intercept = 2.77, 95% CI − 0.79–6.33, t = 1.53, *p* = 0.16).

Figure 1 shows the forest plots of the association between anti-MAP abs seroprevalence and T1DM. A positive and significant risk of T1DM was linked to MAP infection by the models (FE: OR 7.47, 95% CI [5.50; 10.14], *p* value < 0.0001; RE: OR 7.92 [4.39; 14.31], *p* < 0.0001); both models had PI = 1.19–52.59 and a medium level of heterogeneity (I^2^ = 61.0% [28.5%; 78.7%]; ${\chi}_{df=12}^{2}=30.73, Qp=0.0002$). Also, the funnel plot of the MAP-related T1DM according to anti-MAP abs did not detect evidence of publication bias as the Eggers' test was not significant (intercept = 2.77, 95% CI − 0.79–6.33, t = 1.53, *p* = 0.16) (Fig. 2).

**Figure 3:**
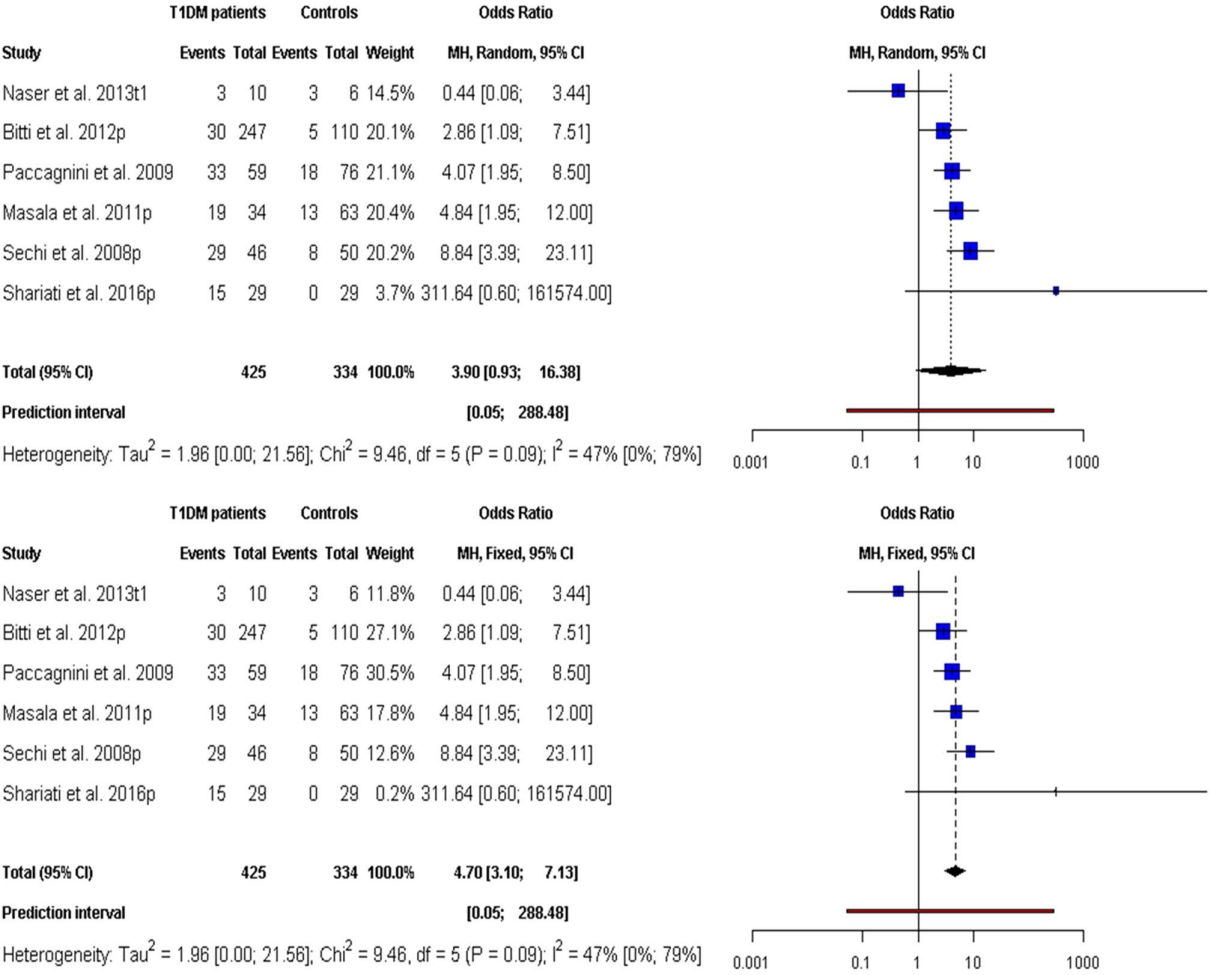
Forest plots for meta-analysis of the risk of MAP (DNA) infections linked with T1DM.

The association between T1DM and MAP as revealed by MAP DNA assessment using PCR assays is presented in Fig. 3. MAP was significantly associated with T1DM with an OR value of 4.70 (95% CI 3.10–7.13, *p* value < 0.0001) and OR 3.90 (95% CI 0.93–16.38, *p* value = 0.06) by FE and RE, respectively. The PI of the MAP DNA FE/RE model ranged from 0.10 to 255.95. Also, a moderate heterogeneity (I^2^ = 47.2%, 95% CI 0.0–79.1%; ${\chi}_{df=5}^{2}=9.46,$ Qp-value = 0.092) was observed in the models.

**Figure 4:**
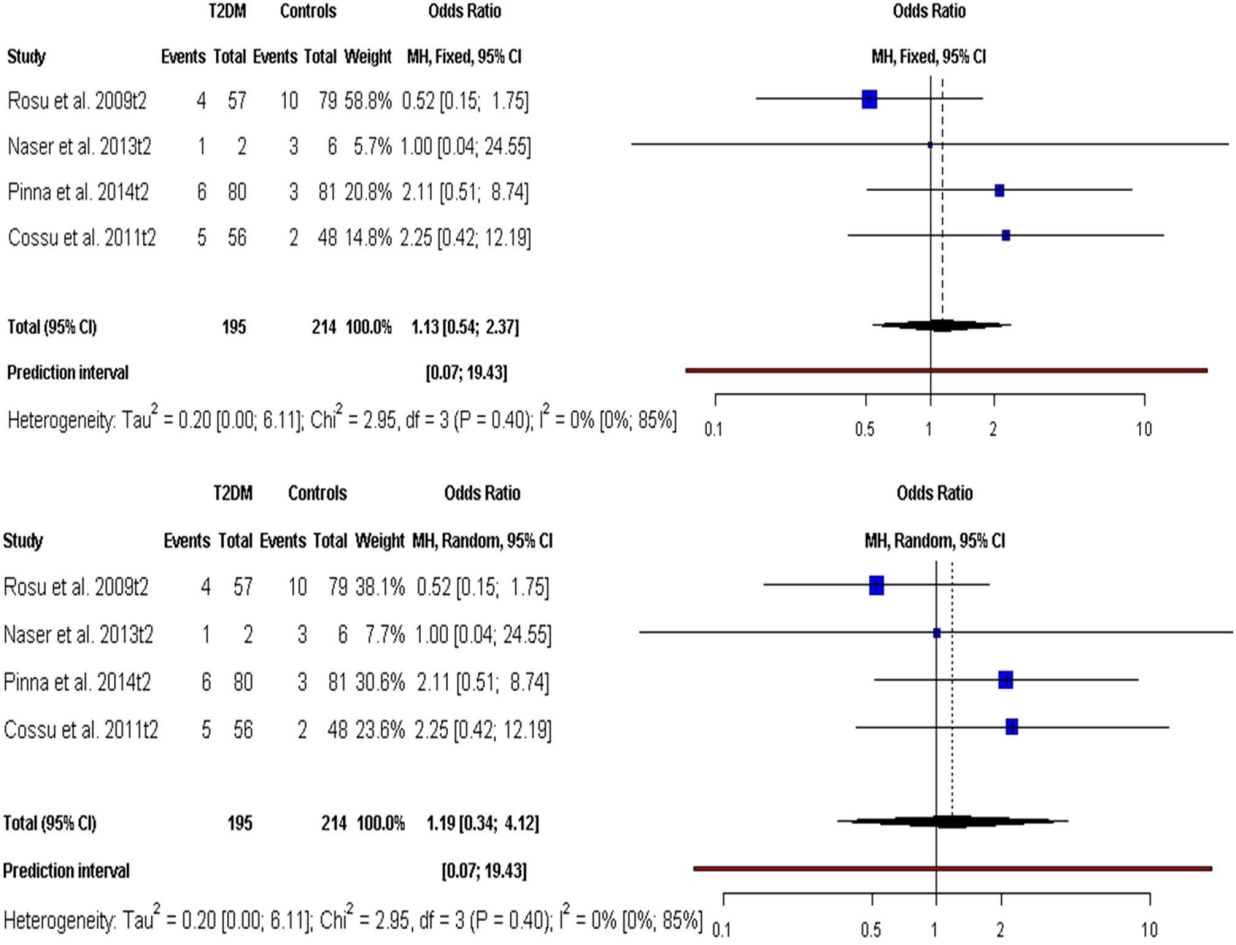
Forest plots for the association of MAP infections with T2DM.

Figure 4 depicts the association between MAP and T2DM. The FE model revealed an OR value greater than 1 of T2DM due to MAP infections (OR 1.13, 95% CI 0.54–2.37, *p* value = 0.74) as well as the RE (OR 1.19; 95% CI 0.34–4.12, *p* value = 0.69; I^2^ = 0.0%) but not statistically significant. Also, the models had no heterogeneity (I^2^ = 0.0%, 95% CI 0.0–84.7%; ${\chi}_{df=3}^{2}=2.95, Qp= 0.40)$. However, the PIs (0.09–15.29) accompanying the models suggest as more studies available, there is possibility that there will be strong association between MAP and T2DM.

Table 2 presents the sensitivity analyses of the 3 meta-analyses. The leave-one-out sensitivity analysis depicts a robust meta-analysis in all cases. While the sensitivity analysis in the anti-MAP abs meta-analysis modified the OR with the minimum and maximum value as 6.93 and 9.28 respectively, omission of Shariati et al.^41^ led to a substantial reduction in the heterogeneity (I^2^ = 38.4%, 95% CI 0.0–68.8%, Qp = 0.09) compared to its main-analysis as a potential source of heterogeneity. Similarly, sensitivity modified OR with minimum as 3.22 and the maximum as 5.30 in the MAP DNA meta-analysis; with omission of Naser et al.^54^ considerably reduced the level of heterogeneity compared with its main analysis (I^2^ = 12.3%, 95% CI 0.0–81.8%, Qp = 0.34). No influential study was identified in anti-MAP abs and MAP DNA meta-analyses. In addition, p-curve analysis of anti-MAP abs meta-analysis indicated evidential true association between MAP and T1DM with considerable significant right-skewness test (PFull < 0.001, PHalf < 0.001) and statistical power (94%, 95% CI 84.3–98.2%) (Table S2). However, Rosu et al.^52^ was identified as potential influential study in T2DM meta-analysis as its omission yielded OR 2.00 (95% CI 0.97–4.14, *p* = 0.06; I^2^ = 0.0%, 95% CI 0.0–89.6%; Qp = 0.90).

**Table 2:** Sensitivity analysis of T1D (anti-MAP abs, MAP DNA) and T2D meta-analyses of MAP-related diabetes.

	OR	*p* value	PI	I^2^-statistic [95 CI]	Q-statistic; *d.f*	*Qp*-value
**T1D anti-MAP abs sensitivity**						
**T1D anti-MAP abs main analysis**	7.92 [4.39; 14.31]	< 0.0001	[1.19; 52.59]	61.0% [28.5%; 78.7%]	30.73; 12	0.002
Study omitted						
Bitti et al. 2012	7.83 [4.07; 15.06]	< 0.0001	[1.03; 59.64]	63.7% [32.6%; 80.5%]	30.34; 11	0.0014
Masala et al. 2011	8.23 [4.31; 15.65]	< 0.0001	[1.10; 61.31]	64.0% [33.1%; 80.6%]	30.53; 11	0.0013
Masala et al. 2012	7.80 [4.07; 14.93]	< 0.0001	[1.03; 60.99]	64.2% [33.6%; 80.7%]	30.73; 11	0.0012
Pinna et al. 2013	8.05 [4.24; 15.31]	< 0.0001	[1.06; 60.99]	64.2% [39.8%; 81.3%]	30.73; 11	0.0012
Pinna et al. 2014	8.17 [4.29; 15.58]	< 0.0001	[1.09; 61.31]	64.1% [33.3%; 80.6%]	30.61; 11	0.0013
Sechi et al. 2008	7.13 [3.96; 12.85]	< 0.0001	[1.18; 43.28]	58.2% [20.8%; 78.0%]	26.34; 11	0.0058
Masala et al. 2014	8.76 [4.71; 16.28]	< 0.0001	[1.31; 58.42]	57.9% [20.1%; 77.8%]	26.12; 11	0.0062
Rosu et al. 2009	8.16 [4.24; 15.69]	< 0.0001	[1.09; 61.75]	64.1% [33.3%; 80.7%]	30.63; 11	0.0013
Noli et al. 2021	7.91 [4.15; 15.07]	< 0.0001	[1.04; 60.16]	64.1% [33.4%; 80.7%]	30.66; 11	0.0012
Niegowska et al. 2016b	6.93 [3.94; 12.19]	< 0.0001	[1.25; 38.31]	54.0% [11.6%; 76.0%]	23.89; 11	0.0132
Niegowska et al. 2016zn	7.65 [4.00; 14.62]	< 0.0001	[1.02; 57.29]	62.8% [30.6%; 80.1%]	29.57; 11	0.0019
Shariati et al. 2016	9.28 [5.70; 15.09]	< 0.0001	[2.28; 37.69]	38.4% [0.0%; 68.8%]	17.86; 11	0.0850
Cossu et al. 2011	7.39 [3.98; 13.72]	< 0.0001	[1.08; 50.60]	61.5%[27.7%; 79.4%]	28.54; 11	0.0027
**T1D MAP DNA sensitivity**						
**T1D MAP DNA main analysis**	3.90 [0.93; 16.38]	0.06	[0.10; 255.95]	47.2% [0.0%; 79.1%]	9.46; 5	0.09
Study omitted						
Bitti et al. 2012	4.28 [0.56; 32.52]	0.12	[0.02; 1180.01]	53.7% [0.0%; 82.9%]	8.64; 4	0.07
Masala et al. 2011p	3.76 [0.49; 29.23]	0.15	[0.01; 1117.13]	57.3% [0.0%; 84.2%]	9.37; 4	0.05
Sechi et al. 2008p	3.22 [0.47; 22.24]	0.17	[0.01; 782.31]	40.3% [0.0%; 78.0%]	6.70; 4	0.15
Naser et al. 2013t1	5.30 [1.75; 16.02]	0.014	[0.15; 183.92]	12.3% [0.0%; 81.8%]	4.56; 4	0.34
Shariati et al. 2016	3.63 [1.12; 11.79]	0.04	[0.17; 77.62]	47.3% [0.0%; 80.7%]	7.58; 4	0.11
Paccagnini et al. 2009	3.93 [0.50; 30.89]	0.1392	[0.01; 1171.34]	57.6% [0.0%; 84.3%]	9.44; 4	0.05
**T2D sensitivity**						
**T2D main analysis**	1.19 [0.34; 4.12]	0.69	[0.09; 15.29]	0.0% [0.0%; 84.7%]	0.95; 3	0.4
Study omitted						
Pinna et al. 2014t2	0.92 [0.12; 7.14]	0.88	[0.00; 4043.22]	0.0%[0.0%; 89.6%]	1.92; 2	0.38
Naser et al. 2013t2	1.23 [0.15; 10.33]	0.72	[0.0001; 20,080.89]	32.1% [0.0%; 92.9%]	2.95; 2	0.23
Rosu et al. 2009t2	2.00 [0.97; 4.14]	0.06	[0.15; 26.73]	0.0% [0.0%; 89.6%];	0.20; 2	0.90 *
Cossu et al. 2011t2	0.97 [0.13; 7.23]	0.96	[0.00; 3713.40]	7.0% [0.0%; 90.3%]	2.15; 2	0.34

* = influential study; OR = odds ratio; PI = prediction interval; d.f. = degree of freedom.

## Discussion

The association between MAP and DM (T1DM and T2DM) was investigated in this study via meta-analysis. The present study found 16 case–control studies conducted in Asia (Iran), Europe (Italy) and North America (USA) that investigated MAP-related DM (Table 1). This suggests that studies on MAP-related DM have not received global attention considering the global burden of DM. There is a need to close the data gap to further assess regional and global burden of MAP-related DM. Regional knowledge of MAP-related DM is very useful in the management of DM and in reducing the burden of MAP-related DM.

Given the various complications that accompany DM and the complexity of the disease, it is important to identify the risk factors involved in the development of DM, with a view to developing effective management strategy. Interestingly, findings from this present study found a substantial and significant association between anti-MAP abs/MAP DNA and T1DM with a medium level of heterogeneity. This further lends credence to various studies that previously implicated MAP as environmental trigger in T1DM. T1DM is known as insulin-dependent diabetes mellitus because of the body’s inability to synthesize insulin, the hormone that regulates blood glucose in the body. This may be characterized by pancreatic β-cell dysfunction^62,63^ or outright destruction of the insulin-secreting beta cells in the pancreas through continuous autoimmune response^64^.

The stronger association of MAP infection with T1DM can be attributed to the continuous T cell mediated autoimmune response of the body against MAP infection, with consequent destructive effect on pancreatic β-cells, which are responsible for insulin production^20,64^. This finding further corroborates the infectious trigger hypothesis, which postulated that MAP may plays a fundamental role in the etiopathology of T1DM^24,65^. Dow^20^ have traced the connection between MAP and T1DM to “molecular mimicry”, in which case protein elements of MAP share sequence and/or conformational elements with the host in such a way that immune responses elicited by the body against the pathogen attack the host cells as well. Dow^54^ has proposed a link between the “mimicry of mycobacterial heat shock protein of MAP (HSP65) and “pancreatic glutamic acid decarboxylase (GAD)”. HSP65 is produced by MAP in response to stress but there exists a homology in the antigenic determinant (epitope) of MAP/human HSP60/65 and pancreatic GAD, which perhaps elicits production of “anti-GAD” antibodies that subsequently destroy the pancreas^66^, the site from which insulin is produced. Hence, generation of “anti-GAD” antibodies poses a risk for developing T1DM.

Furthermore, the findings found PI accompany anti-MAP abs meta-analysis to be much narrower (Fig. 1) compared with MAP DNA analysis (Fig. 3) in assessing the link between MAP infection and T1DM. Aside from the facts that the anti-MAP abs PI testified that anti-MAP abs confirmed that future studies would implausibly support MAP as a risk agent of T1DM, it showed abs/serologic based assessment of MAP as superior to DNA/PCR based technique in assessing T1DM risk due to MAP infections. Because, while abs can linger in the blood post-clearance of the pathogen yielding positive abs linked test results, MAP clearance would result into a false-negative result in PCR assays targeting MAP DNA. Also, the estimate of immune status or establishment of immune response against MAP by the host cannot be achieved via DNA based diagnostic. However, there was larger level of heterogeneity associated with anti-MAP abs compared with MAP DNA meta-analysis. This can be attributed to differences in control and clinical populations, sample preparation, and experimental conditions among others. Disparity between control and clinical populations in term of age, sex ratio, mean diabetes duration and comorbidity of MAP infection with other autoimmune diseases among others, could possibly explain the observed differences.

Meanwhile, this study could not establish a significant association between MAP infection and T2DM because T2DM is a non-autoimmune disease as previous studies did not detect MAP DNA in the blood of T2DM patients^45,58,67^. Whereas, the PI accompanying the meta-analytic models of the association between MAP and T2DM had a narrower range suggesting a possibility that there may be significant/strong association between MAP and T2DM in the future. Therefore, more data are required to examine the true link between MAP infection and T2DM. T2DM is otherwise referred to as non-insulin dependent diabetes mellitus that results from insulin resistance, a situation in which the insulin produced is not appropriately utilized by the cell, this may sometimes, be coupled with absolute insulin deficit^19^. It is not impossible that MAP-related attack on pancreatic beta-cells and associated dysfunctions can lead to production of insulin species lacking the necessary conformations required for glucose and biomolecules metabolisms. Thus, resulting in hyperglycemia and substantial alteration in the metabolism of biomolecules such as lipids, carbohydrates and proteins^68^. It is worthy of note that 90% of diabetic cases exhibit T2DM^69^ and establishment of etiopathologic agents of T2DM might revolutionize its management. It is possible that MAP-related attack activities on pancreatic beta-cells could trigger mutagenic responses that could impair genes responsible for insulin production either to assume partial expression leading to the release of insulins lacking the necessary conformations required for glucose and biomolecule metabolisms or complete downregulation of the gene expression.

The findings of the sensitivity analyses in this study revealed the robustness of the meta-analyses as well as the considerable significant statistical power of the anti-MAP abs meta-analysis. This suggests evidential association between MAP and T1DM as well as not spurious one. Also, the findings observed a substantially reduced heterogeneity with omission of Shariati et al.^41^ in anti-MAP abs meta-analysis and when Naser et al.^54^ omitted in the MAP DNA meta-analysis. There was high disparity between male (7) and female (27) DM patients in Shariati et al.^41^ and may account for the heterogeneity; otherwise, the characteristics of the clinical population. Similarly, the small population size of clinical population (5 females/7 males) and HCs (5 females/1 male) in Naser et al.^54^ could explain the associated potential heterogeneity (Table 1). Further, Rosu et al.^52^ was identified as potential influential study in T2DM meta-analysis. This suggested that Rosu et al.^52^ had large impact or effect on the overall pooled results in MAP-related T2DM meta-analysis (Fig. 4).

General data scarcity on MAP-related DM studies from most regions is one of the major limitations of this study as well as inability to assess the source(s) of the medium heterogeneity associated with anti-MAP abs assays. Also, the quality of the included studies, represented various sources or degrees of risk of biases. The time order of MAP infection and onset of DM was not reported in all the studies synthesised. However, number of days MAP was diagnosed after DM was diagnosed was reported in some studies. The causal role of MAP/time order in DM is still begging for further investigation. In conclusion, this study demonstrates important association between MAP and T1DM highlighting the relevance of MAP infection prevention and environmental management as potential measures in controlling T1DM. Also, the present evidence from this study projects possible association between MAP and T2DM in the future as more data becomes available. However, it remains elusive whether MAP triggers T1/T2DM or a mere comorbidity of T1/T2DM’s complications begging future elucidation, especially the time order of MAP infections prior to onset of DM are essentially to establish causal association. Worldwide elucidation and establishment of etiopathologic agents’ roles in T2DM would indubitably revolutionize its management. Epidemiological activities to fill the global/regional data gaps on MAP-related T1DM and T2DM are advocated in order to assess the burden of MAP-related DM and improve their clinical management.

## Supplementary Information

Supplementary Information.
