# Supplementary material for: Systematic and meta-analysis of Mycobacterium avium subsp. paratuberculosis related type 1 and type 2 diabetes mellitus
Source: Sci Rep. 2022 Mar 17;12:4608. doi: 10.1038/s41598-022-08700-4 (PMC8930973; doi:10.1038/s41598-022-08700-4)
Supplement: Supplementary file 1 — Supplementary Information. [file 41598_2022_8700_MOESM1_ESM.docx]

**Systematic and meta-analysis of *Mycobacterium avium subsp.* *paratuberculosis* related type 1 and type 2 diabetes mellitus**

**Running title: *M. avium subsp.* *paratuberculosis* contributes to the risk of diabetes**

Temitope C. Ekundayo^1,2*^; Ayodeji O. Falade^3^; Bright E. Igere^4^, Chidozie D. Iwu^5;^ Mary A. Adewoyin^6^; Tosin A. Olasehinde^7,8^; and Oluwatosin A. Ijabadeniyi^1^

*^1^Department of Biotechnology and Food Science, Durban University of Technology, Steve Biko Campus, Health Services, 121 Steve Biko Rd, Musgrave, Berea, 4001, Durban, South Africa*

*^2^Department of Biological Sciences, University of Medical Sciences, Ondo City, Ondo State, Nigeria*

*^3^Biotechnology, Computational Biochemistry and Phytomedicine Research Group, Department of Biochemistry, University of Medical Sciences, Ondo City, Ondo State, Nigeria*

*^4^Department of Microbiology and Biotechnology, Western Delta University, Oghara, Delta State, Nigeria*

*^5^School of Health Systems and Public Health, Faculty of Health Sciences, University of Pretoria, Pretoria, South Africa*

*^6^Department of Biological Sciences, Anchor University, Ayobo Road, Ipaja, Lagos*

*^7^Nutrition and Toxicology Division, Food Technology Department, Federal Institute of Industrial Research Oshodi, Lagos, Nigeria,*

*^8^Discipline of Microbiology, School of Life Sciences University of Kwazulu-Natal, Westville campus, Durban, South Africa*

**Corresponding address: temitopee@dut.ac.za*

**Full search strategy**

**1. Google scholar**

Boolean/Phrase:

allintitle: paratuberculosis T1DM OR diabetes OR diabetic OR T2DM -review -case -cases -animal -mice -mouse

**2. PubMed**

Boolean/Phrase:

paratuberculosis[Title] AND (T1DM[Title] OR diabet*[Title] OR T2DM[Title])

Filters applied: Journal Article.

**3. Web of Science Core Collection**

Boolean/Phrase:

paratuberculosis AND (T1DM OR diabet* OR T2DM) (Title)

Refined By: Document Types: Articles

**4. Ebscohost**

Boolean/Phrase:

TI paratuberculosis AND (T1DM OR diabet* OR T2DM)

Searching: Academic Search Complete, Agricola, AHFS Consumer Medication Information, APA PsycBooks, APA PsycInfo, APA PsycTests, AtlaSerials, Religion Collection, Audiobook Collection (EBSCOhost), Business Source Complete, CAB Abstracts, CINAHL with Full Text, Communication & Mass Media Complete, eBook Collection (EBSCOhost), ERIC, Fish, Fisheries & Aquatic Biodiversity Worldwide, Global Health, GreenFILE, Health Source - Consumer Edition, Health Source: Nursing/Academic Edition, Library, Information Science & Technology Abstracts, MasterFILE Premier, MasterFILE Reference eBook Collection, MEDLINE, MEDLINE with Full Text, Newspaper Source, OpenDissertations, Regional Business News, SocINDEX with Full Text, SPORTDiscus with Full Text, Teacher Reference Center

**5. ProQuest**

Boolean/Phrase:

ti(paratuberculosis AND (T1DM OR diabet* OR T2DM))

Additional limits - Document type: Article

**6. Scopus**

Boolean/Phrase:

TITLE ( ( paratuberculos* AND ( t1dm OR diabet* OR t2dm ) ) OR ( 'johne’s AND disease' AND ( t1dm OR diabet* OR t2dm ) ) ) AND ( LIMIT-TO ( DOCTYPE , "ar" ) )

Database search: WOS (16) + Scopus (19) + PubMed (18) + ProQuest (22) + EBSCOhost (21) + Google Scholar (29)

= 125 **(Total documents)**

Record title/abstract screened for inclusion (n = 29)

**Excluded documents (n = 4):**

- Hypothesis with no data (3/4)
- No relevant data (1/4)

Final articles:

- 2 prevalence studies;
- 16 case-control studies
- T1DM &T2DM

Duplicate removed

(n = 96)

Total full-text reviewed (n = 22)

Record removed (n= 7):

- Theses (4/7)
- Review (2/7)
- Book chapter (1/7)

**Identification**

**Screening**

**Eligibility**

**Included**

**Figure S1. Schematic diagram for selecting articles on MAP-related diabetes.**

T1DM = Type-1 diabetes mellitus & T2DM = Type-2 diabetes mellitus.

Table S1. Quality assessment of the included articles.

| **Quality category** | **Noli et al. 2021** | **Bo et al. 2019** | **Niegowska et al. 2016** | **Shariati et al. 2016** | **Pinna et al. 2014** | **A Cossu et al. 2013** | | **Naser et al. 2013** | | **Pinna et al. 2013** | | **Bitti et al. 2012** | | **A Cossu et al. 2011** | | **Masala et al. 2011** | | **Masala et al. 2014** | | **Paccagnini et al. 2009** | | **Rosu et al. 2009** | **Sechi et al. 2008a** | **Sechi et al. 2008b** | **%16 articles** |
| --- | --- | --- | --- | --- | --- | --- | --- | --- | --- | --- | --- | --- | --- | --- | --- | --- | --- | --- | --- | --- | --- | --- | --- | --- | --- |
| Selection |  |  |  |  |  |  | |  | |  | |  | |  | |  | |  | |  | |  |  |  |  |
| 1) Is the case definition adequate?: ___ | |  |  |  |  |  | |  | |  | |  | |  | |  | |  | |  | |  |  |  |  |
| a) Yes, with independent validation (one star) | 2 | 0 | 2 | 0 | 2 | 0 | | 0 | | 2 | | 0 | | 0 | | 0 | | 2 | | 0 | | 0 | 2 | 0 | **37.50** |
| b) Yes, e.g., record linkage or based on self report | 1 | 1 | 0 | 1 | 0 | 1 | | 1 | | 0 | | 1 | | 1 | | 1 | | 0 | | 1 | | 1 | 0 | 1 | **68.75** |
| c) No description | 0 | 0 | 0 | 0 | 0 | 0 | | 0 | | 0 | | 0 | | 0 | | 0 | | 0 | | 0 | | 0 | 0 | 0 | **0.00** |
| 2) Representativeness of the cases: ___ | |  |  |  |  |  | |  | |  | |  | |  | |  | |  | |  | |  |  |  |  |
| a) Consecutive or obviously representative series of cases (one star) | 2 | 2 | 2 | 0 | 2 | 2 | | 2 | | 2 | | 2 | | 2 | | 2 | | 2 | | 2 | | 2 | 2 | 2 | **93.75** |
| b) Potential for selection biases or not stated | 0 | 0 | 0 | 1 | 0 | 0 | | 0 | | 0 | | 0 | | 0 | | 0 | | 0 | | 0 | | 0 | 0 | 0 | **6.25** |
| 3) Selection of controls: __ |  |  |  |  |  |  | |  | |  | |  | |  | |  | |  | |  | |  |  |  |  |
| a) Community controls (one star) | 0 | 0 | 0 | 0 | 0 | 2 | | 2 | | 0 | | 0 | | 0 | | 0 | | 0 | | 0 | | 0 | 2 | 0 | **18.75** |
| b) Hospital controls | 1 | 1 | 1 | 0 | 1 | 0 | | 0 | | 1 | | 1 | | 1 | | 1 | | 1 | | 1 | | 1 | 0 | 0 | **68.75** |
| c) No description | 0 | 0 | 0 | 0 | 0 | 0 | | 0 | | 0 | | 0 | | 0 | | 0 | | 0 | | 0 | | 0 | 0 | 0 | **0.00** |
| 4) Definition of controls: ____ |  |  |  |  |  |  | |  | |  | |  | |  | |  | |  | |  | |  |  |  | **0.00** |
| a) No history of disease (endpoint) (one star) | 2 | 2 | 2 | 2 | 2 | 2 | | 2 | | 2 | | 2 | | 2 | | 2 | | 2 | | **2** | | 2 | 2 | 2 | **100.00** |
| b) No description of source | 0 | 0 | 0 | 0 | 0 | 0 | | 0 | | 0 | | 0 | | 0 | | 0 | | 0 | | 0 | | 0 | 0 | 0 | **0.00** |
| Comparability |  |  |  |  |  |  | |  | |  | |  | |  | |  | |  | |  | |  |  |  | **0.00** |
| 1) Comparability of cases and controls on the basis of the design or analysis controlled for confounders: | | | | | | |  | |  | |  | |  | |  | |  | |  | |  |  |  |  | **0.00** |
| □ The study controls for age (one star) | 2 | 2 | 2 | 2 | 2 | 2 | | 0 | | 2 | | 0 | | 2 | | 2 | | 2 | | 1 | | 1 | 0 | 2 | **81.25** |
| □ Study controls for other factors (list)____ (one star) | 2 | 1 | 2 | 0 | 0 | 0 | | 0 | | 1 | | 0 | | 0 | | 0 | | 0 | | 0 | | 0 | 0 | 0 | **25.00** |
| □ Cohorts are not comparable on the basis of the design or analysis controlled for confounders | 0 | 0 | 0 | 0 | 0 | 0 | | 0 | | 0 | | 0 | | 0 | | 0 | | 0 | | 0 | | 0 | 0 | 0 | **0.00** |
| Exposure |  |  |  |  |  |  | |  | |  | |  | |  | |  | |  | |  | |  |  |  | **0.00** |
| 1) Ascertainment of exposure: ______________ | |  |  |  |  |  | |  | |  | |  | |  | |  | |  | |  | |  |  |  | **0.00** |
| a) Secure record (e.g., surgical record) (one star) | 2 | 2 | 2 | 2 | 2 | 2 | | 2 | | 2 | | 2 | | 2 | | 2 | | 2 | | 2 | | 2 | 2 | 2 | **100.00** |
| b) Structured interview where blind to case/control status (one star) | 0 | 0 | 0 | 0 | 0 | 0 | | 0 | | 0 | | 0 | | 0 | | 0 | | 0 | | 0 | | 0 | 0 | 0 | **0.00** |
| c) Interview not blinded to case/control status | 0 | 0 | 0 | 0 | 0 | 0 | | 0 | | 0 | | 0 | | 0 | | 0 | | 0 | | 0 | | 0 | 0 | 0 | **0.00** |
| d) Written self report or medical record only | 0 | 0 | 0 | 0 | 0 | 0 | | 0 | | 0 | | 0 | | 0 | | 0 | | 0 | | 0 | | 0 | 0 | 0 | **0.00** |
| e) No description | 0 | 0 | 0 | 0 | 0 | 0 | | 0 | | 0 | | 0 | | 0 | | 0 | | 0 | | 0 | | 0 | 0 | 0 | **0.00** |
| 2) Same method of ascertainment for cases and controls: _______________ | | | |  |  |  | |  | |  | |  | |  | |  | |  | |  | |  |  |  | **0.00** |
| □ Yes (one star) | 2 | 2 | 2 | 2 | 2 | 2 | | 2 | | 2 | | 2 | | 2 | | 2 | | 2 | | 2 | | 2 | 2 | 2 | **100.00** |
| □ No | 0 | 0 | 0 | 0 | 0 | 0 | | 0 | | 0 | | 0 | | 0 | | 0 | | 0 | | 0 | | 0 | 0 | 0 | **0.00** |
| 3) Non-response rate: ______________ |  |  |  |  |  |  | |  | |  | |  | |  | |  | |  | |  | |  |  |  | **0.00** |
| a) Same rate for both groups (one star) | 0 | 0 | 0 | 0 | 0 | 0 | | 0 | | 0 | | 0 | | 0 | | 0 | | 0 | | 0 | | 0 | 2 | 2 | **12.50** |
| b) Non-respondents described | 0 | 0 | 0 | 0 | 0 | 0 | | 0 | | 0 | | 0 | | 0 | | 0 | | 0 | | 0 | | 0 | 0 | 0 | **0.00** |
| c) Rate different between cases and controls with no description | 0 | 0 | 0 | 0 | 0 | 0 | | 0 | | 1 | | 0 | | 0 | | 0 | | 0 | | 0 | | 0 | 0 | 0 | **6.25** |
|  | 16 | 13 | 15 | 10 | 13 | 13 | | 11 | | 15 | | 10 | | 12 | | 12 | | 13 | | 11 | | 11 | 14 | 13 |  |

one star = 2; quality perceived < one star = 1; absence/not Applicable = 0.

**Table S2: Pcurve analysis of anti-MAP abs meta-analysis of MAP-related T1D.**

|  |  | Full Curve | |  | Half Curve | |  | | Evidential Value | | | Power Estimate | |
| --- | --- | --- | --- | --- | --- | --- | --- | --- | --- | --- | --- | --- | --- |
|  | pBinomial | zFull | 𝑝Full |  | zHalf | pHalf | |  | | present | Absent |  |  |
| Right-Skewness Test | 0.003 | -8.294 | <0.001 |  | -7.687 | <0.001 | |  | | yes | No | 94% (84.3% **–** 98.2%) | |
| Flatness Test | 0.982 | 5.264 | >0.999 |  | 7.063 | >0.999 | |  | |  |  |  |  |
